# Design of an α-helical antimicrobial peptide with improved cell-selective and potent anti-biofilm activity

**DOI:** 10.1038/srep27394

**Published:** 2016-06-08

**Authors:** Shi-Kun Zhang, Jin-wen Song, Feng Gong, Su-Bo Li, Hong-Yu Chang, Hui-Min Xie, Hong-Wei Gao, Ying-Xia Tan, Shou-Ping Ji

**Affiliations:** 1 Department of Tissue Engineering, Beijing Institute of Transfusion Medicine, Beijing, China; 2 Department of Paediatrics, General Hospital of the PLA Rocket Force, Beijing, China; 3 Department of Rehabilitation Center, General Hospital of the PLA, Beijing, China

## Abstract

AR-23 is a melittin-related peptide with 23 residues. Like melittin, its high α-helical amphipathic structure results in strong bactericidal activity and cytotoxicity. In this study, a series of AR-23 analogues with low amphipathicity were designed by substitution of Ala1, Ala8 and Ile17 with positively charged residues (Arg or Lys) to study the effect of positively charged residue distribution on the biological viability of the antimicrobial peptide. Substitution of Ile17 on the nonpolar face with positively charged Lys dramatically altered the hydrophobicity, amphipathicity, helicity and the membrane-penetrating activity against human cells as well as the haemolytic activity of the peptide. However, substitution on the polar face only slightly affected the peptide biophysical properties and biological activity. The results indicate that the position rather than the number of positively charged residue affects the biophysical properties and selectivity of the peptide. Of all the analogues, A(A1R, A8R, I17K), a peptide with Ala1-Arg, Ala8-Arg and Ile17-Lys substitutions, exhibited similar bactericidal activity and anti-biofilm activity to AR-23 but had much lower haemolytic activity and cytotoxicity against mammalian cells compared with AR-23. Therefore, the findings reported here provide a rationalization for peptide design and optimization, which will be useful for the future development of antimicrobial agents.

Antimicrobial peptides (AMPs) have been isolated and characterized from a wide range of animal, plant and bacterial species and are known to play important roles in the host defence system and innate immunity of all species^1^,^2^,^3^,^4^. AMPs are effective at low micromolar concentrations against a broad range of microorganisms, including in many cases those resistant to traditional antibiotics^5^. Unlike traditional antibiotics that kill or inhibit the growth of bacteria by targeting various biosynthetic processes in growing bacteria, including the synthesis of proteins, RNA, DNA, peptidoglycan, and folic acid^5^,^6^,^7^,^8^, AMPs can destabilize and compromise the physical integrity of the bacterial membrane and therefore are unlikely to evoke bacterial resistance^9^.

Despite the diversity in their structure and amino acid sequences, AMPs are defined as short (10–50 amino acids) peptides possessing an overall positive charge (in general, +2 to +9) and a large percentage (≥30%) of hydrophobic amino acids^3^. These properties permit the peptides to fold into amphipathic conformations upon contact with cell membranes, and the positively charged polar face help the molecules bind to the biomembrane via electrostatic interaction with the negatively charged head groups of phospholipids. Then, the nonpolar face of the peptides allows insertion into the membrane through hydrophobic interactions, causing increased permeability and loss of barrier function of target cells^4^,^10^,^11^. However, therapeutic applications of these AMPs have been hindered by their toxicity or ability to lyse eukaryotic cells. To resolve this obstacle, numerous structure-function studies on both natural and synthetic antimicrobial peptides have been employed to improve AMP activity against the pathogen of interest and reduce toxicity at the therapeutic dose^12^. Among them, melittin, which contains 26 amino acid residues, is one of the most widely studied^13^,^14^,^15^. Melittin is isolated from honeybee (*Apis mellifera*) venom and exhibits strong lytic activity against both eukaryotic and prokaryotic cells^16^,^17^.

An amphipathic nature that segregates basic and hydrophobic residues into a polar and a nonpolar face is recognized as a prerequisite for α-helical AMP activity. However, perfect amphipathicity has often resulted in a simultaneous increase in the bactericidal activity and cytotoxicity^18^. Recently, several studies have suggested that substitution of the nonpolar face with positively charged residues to disrupt the α-helical amphipathic structure appears to be related to reduced haemolytic activity while retaining antimicrobial activity similar to that of the equivalent peptide with no substitutions^19^,^20^,^21^,^22^.

Previous studies have reported that two frog skin-derived antimicrobial peptides, AR-23 from *Rana tagoi*^23^ and RV-23 from *Rana draytonii*^24^, exhibited broad-spectrum growth inhibitory activity against Gram-positive and Gram-negative bacteria^25^. AR-23 exhibits high sequence identity with melittin. The two peptides are composed of 23 amino acids. RV-23 possesses three more positively charged residues compared with AR-23, and the positively charged residues are mainly distributed on its polar face, except for Lys17 on the nonpolar face, which may be the reason why the toxicity of AR-23 is much higher than that of RV-23 against human red blood cells. In this study, AR-23 was utilized as the framework to study the effects of the number and distribution of positively charged residues on its biophysical properties and biological activities through positively charged residue substitution. The peptides were first characterized for their secondary structure in solution (water) and membrane-mimicking environments (sodium dodecyl sulphate [SDS] and trifluoroethyl alcohol [TFE], respectively). Furthermore, the antimicrobial activity against three Gram-positive and three Gram-negative bacteria and the anti-biofilm activity of peptides were subsequently measured. Moreover, the haemolytic property and the cytotoxicity to L929 cells were also determined. Flow cytometry and confocal microscopy were employed to investigate the potential membrane destruction mechanisms of the peptides. The results indicated that the distribution of positively charged residues on AR-23 determined its cell selectivity.

## Results

### Peptide design

**Figure 1:**
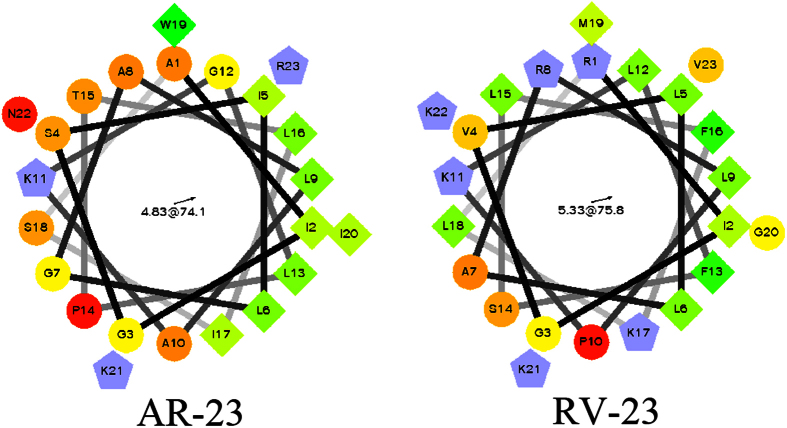
Helical wheel projections of AR-23 and RV-23. By default the output presents the hydrophilic residues as circles, hydrophobic residues as diamonds, potentially negatively charged as triangles, and potentially positively charged as pentagons. Hydrophobicity is color coded as well: the most hydrophobic residue is green, and the amount of green is decreasing proportionally to the hydrophobicity, with zero hydrophobicity coded as yellow. Hydrophilic residues are coded red with pure red being the most hydrophilic (uncharged) residue, and the amount of red decreasing proportionally to the hydrophilicity. The potentially charged residues are light blue.

In this study, the amphipathic α-helical antimicrobial peptide AR-23 was used as a framework to design a series of analogues. To study the effect of positively charged residue distribution on the biological viability of the antimicrobial peptide, single residue at position 1, 8 and 17 of AR-23 were replaced by Arg, Arg and Lys, respectively, which correspond to the positively charged residues of RV-23. The obtained analogues were termed A(A1R), A(A8R) and A(I17K), respectively (Fig. 1). Analogues with two or three residues substitutions were also designed by replacing residues at position (1, 8), (1, 17), (8, 17) and (1, 8, 17) of AR-23 (termed A(A1R, A8R), A(A1R, I17K), A(A8R, I17K) and A(A1R, A8R, I17K), respectively) to explore the effect of the number of positively charged residues on the biological viability of the antimicrobial peptide. To eliminate the influence of Lys at position 17, an Arg residue was introduced to position 17 of AR-23 to create a new analogue named A(I17R) or introduced to position 17 of A(A1R, A8R) to create a new analogue named A(A1R, A8R, I17R).The sequences and characteristics of these analogues are listed in Table 1. The close agreement between the measured and theoretical molecular weights of the peptides indicates that the compounds were synthesized to the desired specifications.

### Circular dichroism (CD) spectroscopic studies of peptides

To determine the secondary structure of peptide analogues in different environments, CD spectra of the peptides were measured under neutral conditions (water), in 50% TFE and in 30 mM SDS. As shown in Fig. S1, all the peptide analogues exhibited a typical α-helix spectrum with double minima at 208 nm and 222 nm in 50% TFE and 30 mM SDS, whereas unordered conformations were observed in water. The α-helical content of AR-23 was 57% in 30 mM SDS (Table 2). Substitution at any one of positions 1, 8 or 17 with positively charged residues decreased the α-helical content, and A(I17K) exhibited the lowest α-helical content of the four analogues. The α-helical content of A(A1R, I17K) was between A(A1R) and A(I17K) and that of A(A8R, I17K) was between A(A8R) and A(I17K); however, the α-helical content of A(A1R, A8R) was lowest among the analogues. In 50% TFE, all analogues except A(A8R) exhibited a significantly decreased α-helical content compared with AR-23, and A(A1R, I17K) exhibited the lowest α-helical content followed by A(A1R, A8R, I17K) and A(A8R, I17K). The α-helical content of the two or three substitution analogues was reduced compared with that of any single substitutions. Generally, the α-helical content of the peptides in 50% TFE was lower than that in 30 mM SDS, and A(A1R, A8R, I17K) exhibited the highest difference in α-helical content between the two buffers.

### Haemolytic activity and cytotoxicity of peptides

**Figure 2:**
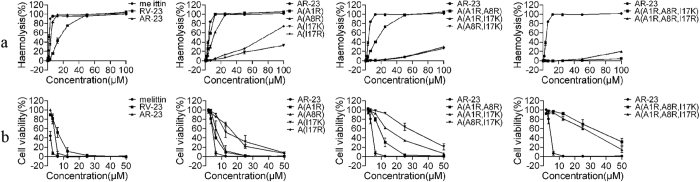
Toxicity of peptides against mammalian cells. (**a**). Haemolytic activity of peptides against human erythrocytes. (**b**). Toxicity of the peptides against L929 cells determined by MTT assay.

The haemolytic activities of the peptides against human erythrocytes were determined as an indication of their toxicity towards mammalian cells. The haemolytic activities of all peptides are summarized in Fig. 2a. A(A1R) and A(A8R) exhibited slightly lower haemolysis rates than AR-23 (25.9% and 12.9% compared with 37.9%, respectively, at concentrations of 3.13 μM). A(I17K) and A(I17R) exhibited dramatically lower haemolysis rates than AR-23. Even at concentrations of 100 μM, the haemolysis rates of A(I17K) and A(I17R) were only 33% and 74%, respectively. With the increasing number of the positively charged residues, the haemolytic activities of the peptides were reduced. The haemolysis rates of the two residue substitution analogues were reduced compared with those of any single residue substitution analogues, and A(A1R, A8R, I17K) exhibited the lowest haemolysis rate, which was only 4.4% at 100 μM.

To further examine the toxicity of these peptides against mammalian cells, the MTT assay was performed on L929 cells. All the analogues exhibited lower toxicity than AR-23 (Fig. 2b). For example, when peptide concentration was 6.25 μM, the viability of AR-23-treated cells was dramatically reduced to approximately 10%, but all the analogue-treated cells exhibited considerably increased viability. Among them, A(A1R, A8R, I17K) exhibited the lowest toxicity towards L929 cells. At a concentration of 50 μM, the viability of A(I17K)-, A(I17R)-, A(A1R, I17K)-, A(A8R, I17K)- and A(A1R, A8R, I17K)-treated cells was 7.9%, 6.6%, 6.3%, 21.1% and 31.4%, respectively, whereas other analogues killed almost all the cells. The results also indicated that substitution at Ile17 of AR-23 with a positively charged residue can greatly reduce the toxicity of the peptides towards the mammalian cells.

### Antimicrobial activity of peptides

The antimicrobial activities of the peptides, indicated by the minimum inhibitory concentrations (MIC), were determined against a range of Gram-negative (Table 3) and Gram-positive bacterial strains (Table 4). The geometric mean of MIC values (GM) from the three strains was calculated to provide an overall evaluation of antimicrobial activity. A(A1R), A(A8R) and A(A1R, A8R) displayed slightly higher antimicrobial activity against the Gram-negative bacteria species compared with AR-23, while A(I17K), A(I17R), A(A1R, I17K), A(A8R, I17K) and A(A1R, A8R, I17K) exhibited lower antimicrobial activity. A(A8R) exhibited a twofold increase in antimicrobial activity compared with parental AR-23 against *S. aureus*. However, A(I17K) exhibited an eightfold decrease in antimicrobial activity, and A(A1R, I17K), A(A8R, I17K) and A(A1R, A8R, I17K) showed sixteenfold decreases in antimicrobial activity. Similar to the results of the toxicity of the peptides to mammalian cells, A(I17R) exhibited a twofold increase in antimicrobial activity compared with A(I17K).

The therapeutic index (TI) is defined as the ratio of the minimum haemolytic concentration (MHC) of peptides to the GM of peptides, and this value was used to evaluate cell selectivity of peptides towards the negatively charged region of bacterial cell membranes over the zwitterionic mammalian cell membranes. All the analogues exhibited an improvement of therapeutic index except A(A1R). A(A1R, A8R, I17K) exhibited the highest therapeutic index, which was 16 and 10.08 against Gram-negative and Gram-positive bacterial strains, respectively. Larger values for the therapeutic index indicate greater antimicrobial specificity, and the therapeutic index of A(A1R, A8R, I17K) was significantly increased against Gram-negative bacteria by 80-fold and Gram-positive bacteria by 12.7-fold compared with AR-23. A(I17K) and A(I17R) had similar therapeutic index values due to the increased toxicity to human erythrocytes of A(I17R) and the enhanced antimicrobial activity. These results indicate that A(A1R, A8R, I17K) exhibited increased cell selectivity towards bacterial cells compared with human erythrocytes, implying a wider therapeutic window.

### Membrane damage induced by the peptides

**Figure 3:**
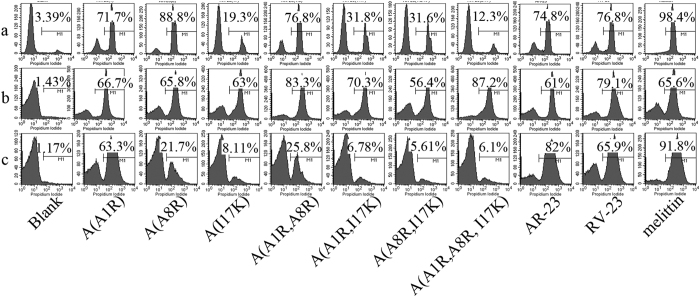
The membrane damage of L929 cells, *E. coli* and *S. aureus* treated by peptides, as measured by an increase in fluorescence intensity of PI. (**a**) L929 cells treated with 25 μM peptides. (**b**) *E. coli* treated with 25 μM peptides. (**c**) *S. aureus* treated with 6.25 μM peptides. The control was processed without peptides.

Propidium iodide (PI) staining of nucleic acids in cells indicates compromised cell membranes structure and cell death. The effect of peptides on L929 cells, *E. coli* and *S. aureus* was probed by incubating the peptide-treated cells with PI followed by flow cytometry (FACS) analysis to determine peptide-induced membrane damage. PI staining of L929 cells (1 × 10^6^) following the treatment of the peptides (25 μM) is presented in Fig. 3a. The peptide-induced damage of L929 cell membranes showed a similar trend to the haemolytic activity and toxicity of the peptides.

The membrane damage of peptides to *E. coli* cells was determined by FACS and was similar to the results of the MICs. The percentage of PI-positive cells with membrane damage by AR-23 was 61% at 25 μM (Fig. 3b). All the analogues of AR-23 exhibited increased membrane damage against *E. coli* except A(A8R, I17K) at this concentration. A(A1R, A8R, I17K) showed the highest potential to damage the *E. coli* membrane (87.2% PI-positive cells). At 6.25 μM, the percentage of PI-positive cells with membrane damage by AR-23 was 82%, and all analogues exhibited decreased membrane damage abilities against *S. aureus* (Fig. 3c). A(A8R, I17K) exhibited the lowest percentage of PI-positive cells (5.61%).

### Membrane-penetrating activity of the peptides

**Figure 4:**
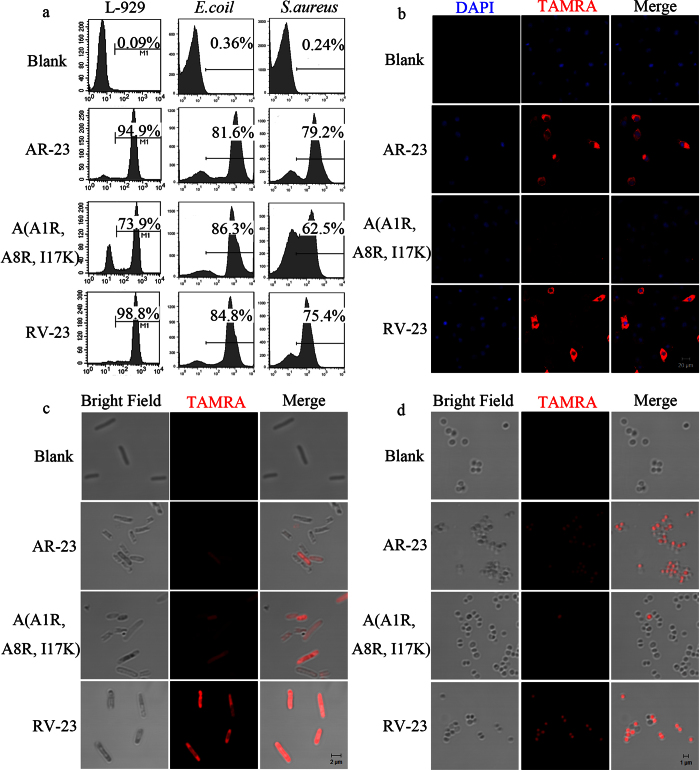
Binding and localization of the TAMRA-labelled peptides to L929 cells, *E. coli* and *S. aureus* as measured by FACS and confocal microscopy. (**a**) Binding of the TAMRA-labelled peptides to L929 cells, *E. coli* and *S. aureus* as measured by FACS. (**b**) Localization of the TAMRA-labelled peptides to L929 cells. (**c**) Localization of the TAMRA-labelled peptides to *E. coli*. (**d**) Localization of the TAMRA-labelled peptides to *S. aureus*.

The interaction of TAMRA-labelled peptides with the membrane of L929 and bacteria cells was investigated by FACS and confocal microscopy. As shown in Fig. 4a, flow cytometry results indicated that 94.9% of TAMRA-labelled AR-23-treated L929 cells exhibited fluorescence, whereas 74% for TAMRA-labelled A(A1R, A8R, I17K)-treated cells exhibited fluorescence. Confocal microscopy revealed that some AR-23-treated L929 cells showed red fluorescence, and most of the signal was focused in the cytoplasm (Fig. 4b). However, only weak red fluorescence signals could be observed on the surface of A(A1R, A8R, I17K)-treated cells. Unlike AR-23, A(A1R, A8R, I17K) at 25 μM could only bind to the surface of L929 cells instead of penetrating into cells.

Regarding bacterial cells, approximately 81.6% and 86.3% of TAMRA-labelled AR-23- and A(A1R, A8R, I17K)-treated *E. coli* cells exhibited fluorescence, respectively (Fig. 4a). However, red fluorescence in TAMRA-labelled A(A1R, A8R, I17K)-treated *S. aureus* cells was considerably reduced compared with TAMRA-labelled AR-23-treated *S. aureus* cells (62.5% vs. 79.2%). The results were consistent with the antibacterial and PI staining studies, indicating that AR-23 and A(A1R, A8R, I17K) had similar binding and penetrating activity against the *E. coli* cell membrane, whereas the A(A1R, A8R, I17K) penetrating activity against *S. aureus* cells was dramatically reduced. Confocal microscopy also indicated that TAMRA-labelled A(A1R, A8R, I17K) exhibited an improved ability to bind and penetrate the *E. coli* cell membrane, which was reversed for the membrane of *S. aureus* cells (Fig. 4c,d).

### Anti-biofilm activity of peptides

**Figure 5:**
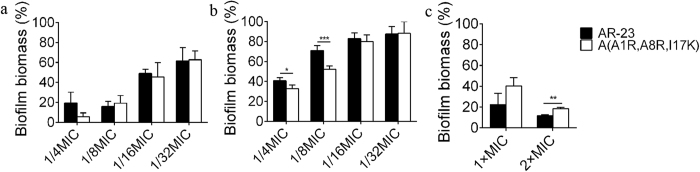
Anti-biofilm activity of the peptides. (**a**) The peptides inhibited the attachment of *A. baumannii*. (**b**) The peptides inhibited the formation of biofilm. (**c**) The peptides dispersed the pre-matured biofilm of *A. baumannii*. *P < 0.05, **P < 0.01, ***P < 0.001.

Biofilm formation is a complex process that generally involves three stages: (1) primary adhesion to surfaces, (2) accumulation of multilayered clusters of cells, and (3) detachment. Experiments were performed to determine the stage of biofilm formation that AMPs disrupt. The MICs of AR-23 and A(A1R, A8R, I17K) against *Acinetobacter baumannii* were 12.5 μM. As shown in Fig. 5a, AR-23 and A(A1R, A8R, I17K) exhibited a dose-dependent inhibition of *A. baumannii* attachment to abiotic surfaces, and A(A1R, A8R, I17K) showed more potential than AR-23. Next, we performed the biofilm formation assay to determine the effects of AMPs on *A. baumannii* biofilm development. As shown in Fig. 5b, AR-23 and A(A1R, A8R, I17K) also displayed a concentration-dependent inhibition of biofilm development. For example, at 1/4 MIC, the inhibition of biofilm formation reached approximately 60%, and A(A1R, A8R, I17K) exhibited increased inhibition. Finally, the preformed *A. baumannii* biofilms on MBEC pegs were used to determine the ability of the AMPs to disperse mature biofilms. AR-23 and A(A1R, A8R, I17K) significantly reduced biofilm mass at the MIC (Fig. 5c). For example, at the MIC, AR-23 and A(A1R, A8R, I17K) could disperse the pre-formed biofilms by 77.6% and 59.6%, respectively.

## Discussion

The emergence of clinical bacterial strains exhibiting resistance to conventional antibiotics has prompted numerous researchers to focus on novel classes of antimicrobial molecules^26^,^27^,^28^. AMPs represent a new potential class of antibiotics as they possess a distinctive antimicrobial mechanism compared with traditional antibiotics^1^,^29^,^30^,^31^,^32^. Although the exact mode of action of antimicrobial peptides has not been established, it has been proposed that the cytoplasmic membrane is the main target of some peptides. Thus, the development of resistance to membrane-active peptides is not expected because this would require substantial changes in the lipid composition of cell membranes of microorganisms. In general, however, therapeutic applications of these peptides have been hindered by several problems, perhaps the most important being toxicity to host cells. To resolve this problem, numerous structure-function studies on both natural and synthetic antimicrobial peptides have been employed to engineer AMPs that are active against the pathogen of interest and have low toxicity at the therapeutic dose^12^.

Physicochemical parameters believed to be important for antimicrobial activity of AMPs have been identified, including net charge, helicity, hydrophobicity, amphipathicity and the angle subtended by the positively charged polar helix face (Φ)^33^. However, it is difficult to estimate the contributions of these parameters to antimicrobial activity and cell selectivity because these parameters are not necessarily independent. In this study, Ala1, Ala8 and Ile17 of AR-23 were systematically substituted with positive charged residues to study the effects of the number and distribution of positively charged residues on the biophysical properties and biological activities of the peptide.

The calculated amphipathicity of the peptides reflected by the μH (mean relative hydrophobic moment) is presented in Table 1. Ala8 on the polar face substituted by positively charged Arg increased the peptide amphipathicity. Ile17 on the nonpolar face substituted by positively charged Lys decreased the peptide amphipathicity, and Ala1 substituted by Arg had no effect on the peptide amphipathicity. However, when Ala1 and Ala8 were both substituted with Arg residues, the peptide amphipathicity was higher than any of them. It is notable that residue 1 appears to fall on the nonpolar face; however, in a membrane surface-localized helical peptide, this reside can re-position itself in the polar regions of the membrane interface by helix “fraying” that is often encountered in the interaction of interfacially localized helical peptide with membranes^34^,^35^,^36^. Perfect amphipathicity has often resulted in a simultaneous increase in the bactericidal activity and cytotoxicity^18^. Disruption of the α-helical amphipathic structure appears to be related to strong antimicrobial activity, reduced haemolytic activity, and hence, an improved AMP therapeutic index^19^,^20^,^21^,^22^. In this study, A(A8R) and A(A1R, A8R) with increased amphipathicity also exhibited reduced haemolytic activity. Amphipathicity is apparently not the most important parameter of these peptides.

The retention time data determined by reversed-phase high-performance liquid chromatography (RP-HPLC) reflects the hydrophobicity difference between peptide analogues. It is well documented that the formation of a hydrophobic-binding domain due to peptide secondary structure can affect peptide interactions with reversed phase matrices. This effect was observed particularly for amphipathic α-helical peptides^37^. Given this preferred binding domain, amphipathic α-helical peptides are considerably more retentive than non-amphipathic peptides with the same amino acid composition. All the analogues in this study had an increased overall number of positive charges residues. Hydrophobicity of analogues should decrease to some degree. Indeed, all the analogues except A(A8R) exhibited decreased retention times ranging from 5.16 to 15.69 min compared with 16.6 min for AR-23 (Table 1). Disruption of the hydrophobic/ nonpolar face by substitution of Ile17 with positively charged Lys significantly reduced the retention time of the peptide (16.6 min vs 8.0 min). However, Ala8 on the polar face substituted by positively charged Arg slightly increased the retention time (16.6 min vs 19.61 min). This finding may be attributed to increased peptide amphipathicity and thus, an increase in the binding of the peptide to the reversed phase matrices. Other analogues with higher calculated amphipathicity had a decreased retention time, which may be due to alteration of the binding domain of the peptide. The result was also in accordance with the α-helical content of peptides in 50% TFE (mimicking the hydrophobic environment). A(A8R) possesses 47% α-helical content compared with 46% of AR-23.

Many studies have demonstrated that the propensity to form an amphipathic α-helix in membrane-mimetic environments is critically important for the membrane-disruptive activity of AMPs^18^. The stabilization energy of peptides forming α-helix structure calculated by InsightII2000 software showed that the secondary structures of the analogues were similar to the parent peptide (Table 1). CD spectra indicate that all the peptides exhibited a random coil in water. In 30 μM SDS and 50% TFE, all peptides exhibited a typical α-helical structure, and increased helical content was observed in 30 μM SDS compared with 50% TFE (Table 2). Previous studies have demonstrated that the helical content was decreased with increased positively charged residues^38^,^39^. Indeed, increasing positively charged residues on the polar or nonpolar face of AR-23 reduced the α-helical content compared with AR-23 except that of A(A8R) in 50% TFE (46% vs. 47%). The α-helical content in 50% TFE appeared in accordance with the peptide retention time, whereas no such tendency was noted in 30 μM SDS. In the two membrane-mimicking environments, disruption of the nonpolar face by substitution of Ile17 with positively charged Lys significantly decreased the α-helical content of the peptide compared with the polar face substituted with a positive charged residue. The Gibbs free energy of peptides partition from water to a membrane interface (ΔG_if_) were calculated by MPEx according to the helicities of peptides in 30 μM SDS and 50% TFE (Table 2). Low ΔG_if_ of peptides means strong membrane partition. Substitution at Ala1 or Ala8 slightly increase the ΔG_if_, while substitution at Ile17 could significantly increase the ΔG_if_, which means that Ile17 substitution would decrease the peptide partition on membrane. A(A1R, A8R, I17K) showed the highest ΔG_if_ (-3.67 kcal/mol) among the analogues according to the helix content of peptides in 50% TFE, which indicated that A(A1R, A8R, I17K) had the lowest ability to bind the eukaryocyte membrane.

It is believed that increasing positively charged residues is beneficial for the initial electrostatic interactions between AMPs and negatively charged bacterial membrane components, thus imposing selectivity^18^,^40^. However, this is not always the case, as our results indicate that A(A1R), A(A8R) and A(A1R, A8R) with increased positively charged residues possessed an increased antimicrobial activity against Gram-negative bacteria, and A(A8R) also exhibited slightly increased antimicrobial activity against Gram-positive bacteria (Table 3). In contrast, other analogues with increased positively charged residues possessed a decreased antimicrobial activity against both Gram-negative and Gram-positive bacteria (Table 4). The results indicate that increasing the peptide amphipathicity by substitution with positively charged residues on the polar face would enhance the peptide antimicrobial activity; however, decreasing the peptide amphipathicity by substitution with positively charged residues on the nonpolar face would have the opposite effect. From these results, we can conclude that the position of positively charged residues in the peptide significantly influences the peptide antimicrobial activity, and obvious correlation is noted between the net charge and the peptide antimicrobial activity. To our surprise, substitution on the nonpolar face dramatically decreased the antimicrobial activity against *S. aureus*; this may be attributed to the specific membrane structure of *S. aureus*.

Obtaining the maximum possible antimicrobial activity with minimum toxicity towards the host is an attractive direction for antimicrobial peptide research and development. The haemolytic activity of the peptides against human erythrocytes was employed as a major measure of peptide toxicity towards higher eukaryotic cells. Biological studies showed that strong haemolytic activity of the peptides generally correlated with high hydrophobicity, high amphipathicity, and high helicity^41^,^42^,^43^,^44^,^45^. Our results indicate that increasing the positive charge appeared to outweigh other factors in the reduction of the haemolytic activity. In contrast to the results of antimicrobial activity testing, all analogues showed a decreased haemolytic activity. A(A1R), A(A8R) and A(A1R, A8R) exhibited slightly decreased haemolytic activity with improved antimicrobial activity. As mentioned above, destruction of the nonpolar face with positively charged residues could dramatically decrease the peptide hydrophobicity, peptide amphipathicity and peptide helicity, and the substitution also significantly decreased the peptide haemolytic activity. For example, at 25 μM, the haemolytic activity of A(A8R) and A(I17K) was 98.3% and 6.4%, respectively. Among all the analogues, A(A1R, A8R, I17K) exhibited the lowest haemolytic activity, which was only 4.4% even at 100 μM. Peptide toxicity to L929 cells was well correlated with the results of haemolytic activity.

The TI is a widely employed parameter to indicate the specificity of antimicrobial reagents, which is calculated by the ratio of MHC (haemolytic activity) and MIC (antimicrobial activity). Thus, larger TI values indicate greater antimicrobial specificity. TI could be increased via one of the following three mechanisms: increasing antimicrobial activity, decreasing haemolytic activity while maintaining antimicrobial activity, or a combination of both increasing antimicrobial activity and decreasing haemolytic activity. The native peptide AR-23 is a peptide with high antimicrobial activity coupled with strong haemolytic activity; hence, its TI is low (0.2 and 0.79 for Gram-negative and Gram-positive bacteria, respectively) (Tables 3 and 4). In this study, by increasing net positive charge, hydrophobicity, amphipathicity, and helicity of AR-23 analogues were changed. The TI of A(A1R, A8R, I17K) against Gram-negative and Gram-positive bacteria significantly increased by 80-fold and 12.7-fold, respectively. Substitution on the nonpolar face of the peptide decreased the antimicrobial activity accompanied by a reduction in haemolytic activity and cytotoxicity. The increased therapeutic index was mainly ascribed to the reduction of haemolytic activity, indicating that the substitution on the nonpolar face mainly affected the haemolytic activity. In consideration of AR-23, which exhibits high sequence identity with melittin, residues at position 1, 8 and 17 of melittin were replaced by Arg, Arg and Lys, which correspond to the positively charged residues of RV-23 to create a new analogue termed M(G1R, V8R, I17R) (Table S1). M(G1R, V8R, I17R) also showed a typical α-helical structure (Fig. S2). Compared with melittin, M(G1R, V8R, I17R) exhibited dramatically reduced cytotoxicity against mammalian cells (Fig. S3), while maintaining a comparable antimicrobial activity (Tables S2, and S3). The TI of M(G1R, V8R, I17R) against Gram-negative and Gram-positive bacteria significantly increased by 101-fold and 64-fold, respectively. These results further highlight that increasing positively charged residues in appropriate positions will improve the peptide TI.

To further investigate the mechanism of selective membrane lytic activity, the fluorescence-labelled peptides were incubated with bacteria or L929 cells and investigated by FACS and confocal microscopy. The results indicate that A(A1R, A8R, I17K) exhibited increased binding or penetrating activity against *E. coli* and significantly lower binding or penetrating activity against L929 cells and *S. aureus* compared with AR-23. The results were strongly in accordance with the antimicrobial activity and cytotoxicity of the two peptides. PI staining assays obtained similar results, indicating that A(A1R, A8R, I17K) had a reduced ability to penetrate the membrane of L929 cells and *S. aureus* cells. These results suggest A(A1R, A8R, I17K) had low cytotoxicity against L929 cells and low antimicrobial activity against *S. aureus*.

Biofilms are microbial communities of sessile microorganisms composed of cells that are embedded in a matrix of extracellular polymeric substances attached to a substratum or interface^46^,^47^. The most alarming aspect of biofilm-related infections is that they are highly (10- to 1,000-fold) resistant to conventional antibiotics that were developed to kill planktonic cells^48^,^49^. Accordingly, the discovery of anti-infective agents that are active against both planktonic microorganisms and biofilms is of great public health significance. In this study, A(A1R, A8R, I17K) inhibited biofilm formation and possessed biofilm dispersal activity against *A. baumannii*.

## Conclusion

In this paper, a series of designed AMPs with an increased net positive charge were synthesized and evaluated for their efficacy against both Gram-negative and Gram-positive bacteria. These peptides were unstructured in water but were folded into an α-helical structure upon interacting with a membrane environment (30 μM SDS and 50% TFE). Substitution in the nonpolar face of the peptide could dramatically decrease the hydrophobicity, amphipathicity, helicity, and membrane-penetrating activity against human cells as well as the haemolytic activity of the peptide, while maintaining or only slightly decreasing the antimicrobial activity. The TI of A(A1R, A8R, I17K) was increased 80-fold against Gram-negative bacteria and 12.7-fold against Gram-positive bacteria compared with the parental AR-23. The results indicate that the position rather than the number of positively charged residues affects the biophysical properties and selectivity of peptides. Therefore, the findings reported here also provide a rationalization for peptide design and optimization, which will be useful for the future development of antimicrobial agents, biomedical coatings and health care applications.

## Materials and Methods

### Materials

The RV-23 and AR-23 peptides and AR-23 analogues were synthesized (Table 1) using the standard Fmoc procedure purified by reverse-phase semipreparative high-performance liquid chromatography as described and were dissolved in water for a 1000 μM stock solution for further use. The purity of the synthetic peptide was greater than 95%. Blood samples from healthy donors were obtained from the Beijing Red Cross Blood Centre. SDS and TFE were from Amresco (USA) and Sigma (USA), respectively, and 3-(4,5-dimethyl-2-thiazolyl)-2,5-diphenyl-2H-tetrazolium bromide (MTT) was from Amresco (USA).

### Circular dichroism (CD) spectropolarimetry of the peptides

CD spectra were recorded on a JASCO J-715 spectropolarimeter (JASCO Inc., Easton, MD, USA) in 0.1 cm pathlength cells under nitrogen at 25 °C. The spectra were recorded between 195 and 250 nm at a peptide concentration of 50 μM in SDS. The percentage of α-helix structure was calculated as^50^:


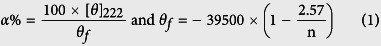


where α is the amount of helix and n is the number of amino acid residues, [*θ*]_222_ is the experimentally observed absolute mean residue ellipticity at 222 nm.

### Wimley−White Interfacial Scale analysis and Sequence analysis

To characterize the binding of peptides to the lipid membrane interface, the free energies of peptide binding from water to the membrane water interface (ΔG_if_) according to the Wimley−White interfacial scale were calculated using the package MPEx^51^. For each peptide, the values were obtained by choosing the interface scale (IF), assuming free N-termini and amidation of the C-termini, and the experimentally determined helicities for different peptides in 30 μM SDS and 50% TFE.

The 3-D structures of AR-23 and its analogues were constructed and the stabilization energy was calculated using InsightII2000 software. The mean relative hydrophobic moment (μH) was performed online using EMBOSS PROTEIN PROPERTIES: http://www.bioinformatics.nl/emboss-explorer/. The helical wheel projection was performed online using the Helical Wheel Projections: http://rzlab.ucr.edu/scripts/wheel/wheel.cgi.

### Antimicrobial activity of the peptides.

The antibacterial activities of the peptides against three Gram-negative bacteria and three Gram-positive bacteria strains were measured using a modified version of the Clinical Laboratory and Standards Institute (CLSI) broth microdilution method as described previously^52^. Three Gram-negative bacteria, *Escherichia coli* (1.8732), *Pseudomonas aeruginosa* (1.2421), and *Klebsiella pneumonia* (1.1736), and three Gram-positive bacteria, *Staphylococcus aureus* (1.8721), *Staphylococcus epidermidis* (1.4260), and *Bacillus subtilis* (1.3376), were purchased from the China General Microbiological Culture Collection Centre (CGMCC). Briefly, bacteria were grown in liquid LB medium (tryptone, 10 g/L, yeast extract, 5 g/L and NaCl, 10 g/L) at 37 °C to mid-log phase, and the bacteria were diluted to 1 × 10^6^ colony-forming units (CFU)/ml. The peptides were serially diluted with phosphate-buffered saline (PBS), and 50 μl diluted peptides were added to 50 μl of the bacterial suspension in 96-well plate (Corning Inc., Lowell, MA, USA). After incubation at 37 °C for 18 to 20 hours, the absorbance of each well was recorded using a multi-well microplate reader (SpectraMax M5; Molecular Devices, Sunnyvale, CA, USA) at 600 nm. The lowest concentration at which the peptide inhibited complete growth of the bacteria was taken as the minimal inhibitory concentration (MIC).

### Haemolytic activity of the peptides

Haemolytic activity of the peptides was assayed by a standard procedure with slight modification^23^. Fresh human erythrocytes were washed three times and resuspended at 1.25% haematocrit in PBS. Forty microlitres PBS-diluted peptide solution was added to a V-bottom 96-well plate (Corning Inc., Lowell, MA, USA), and 160 μl of erythrocytes was added. After incubation at 37 °C for 30 min, the samples were centrifuged, and the absorbance of the supernatant was measured at 450 nm using a multi-well microplate reader (SpectraMax M5; Molecular Devices, Sunnyvale, CA, USA) and compared with the 100% haemolysis caused by 0.1% Triton X-100. The percentage of haemolysis was calculated according to the equation (2):


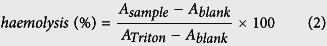


### Cytotoxicity of the peptides

The toxicity of the peptides against L929 mouse fibroblast cells was assessed by a standard MTT assay^24^. L929 mouse fibroblast cells from ATCC (Manassas, VA, USA) were maintained in our laboratory. L929 cells were cultured in RPMI 1640 supplemented with 10% (v/v) FBS, 2 mM L-glutamine, 100 U/ml penicillin and 100 mg/ml streptomycin and maintained in a humidified incubator with 5% CO_2_ at 37 °C. L929 cells (5 × 10^3^cells/well) were seeded in 96-well plates (Corning Inc., Lowell, MA, USA) and incubated overnight. Diluted peptides were added to L929 cells and incubated for 1 hour. Then, 20 μl of MTT (5 mg/ml) solution was added and incubated for 4 hours. The medium was removed, and 150 μl of DMSO was added to each well to dissolve the formazan crystals. The absorbance was measured at 490 nm on a microplate reader (SpectraMax M5; Molecular Devices, Sunnyvale, CA, USA). Cell viabilities were calculated by using the equation (3):


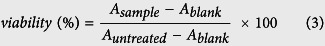


### Bacterial cell membrane damage induced by the peptides

Peptide-induced membrane damage was examined by detection of propidium iodide (PI) influx^53^,^54^. L929 cells (1 × 10^6^ cells) were treated with peptide (final concentration 25 μM) and incubated for 30 min at 37 °C, and the cells were washed with PBS and resuspended in PI solution (final concentration 2 μg/ml). The bacteria were cultured at 37 °C to mid-log phase and then diluted to an OD_600_ = 0.5 in phosphate buffered saline (PBS). Then, AMPs (final concentration for *E. coli* and *S. aureus* was 25 μM and 6.25 μM, respectively) were added to 200 μl bacterial suspension and incubated for 30 min. Bacteria were collected and resuspended in PI solution. The fluorescence signal in treated cells was determined by flow cytometry (Cytomics FC 500, Beckman Coulter).

### Localization and binding of the peptides onto mammalian and bacterial cells

Localization and binding of the peptides onto mammalian and bacterial cells were evaluated using TAMRA-labelled peptides^17^. L929 cells (1 × 10^4^ cells/well) were seeded in a CVG 8-well chamber (NUNC Lab-Tek^TM^). After 24 hours, 6.25 μM of TAMRA-labelled peptide was added to the L929 cells. After incubation for 30 minutes, the cells were washed with PBS and fixed with 4% paraformaldehyde. Then, the cells were stained with the nuclear stain DAPI (final concentration 300 nM) and observed under a confocal scanning laser (Radiance 2100TM, Bio-Rad, Hercules, CA) with an inverted fluorescence microscope (TE300, Nikon, Melville, NY). Typical fluorescence images were obtained at the same time. For flow cytometry detection, L929 cells (1 × 10^6^ cells) were treated with TAMRA-labelled peptide (final concentration was 25 μM), incubated for 30 min at 37 °C, washed with PBS and subjected to flow cytometry (Cytomics FC 500, Beckman Coulter).

Mid-log phase (OD_600_ = 0.5) suspensions of *E. coli* and *S. aureus* were also incubated with TAMRA-labelled peptide solution (final concentration for *E. coli* and *S. aureus* was 25 μM and 6.25 μM, respectively) for 30 min at 37 °C. Then, localization of the peptides on the bacterial cells was observed under a confocal scanning laser (Radiance 2100TM, Bio-Rad, Hercules, CA) with an inverted fluorescence microscope (TE300, Nikon, Melville, NY) and quantified by flow cytometry.

### Inhibition of bacterial initial attachment

Biofilm attachment assays against *Acinetobacter baumannii* were performed using a previously described method with some modifications^55^. *A. baumannii* was a clinical isolates of biofilm-producing bacterial strain. The MICs of AMPs against *A. baumannii* were evaluated as described above. Overnight cultures of *A. baumannii* were washed and resuspended in TSB (tryptic soy broth) to an OD_600_ = 0.05. Aliquots of 100 μl suspended bacteria were added to a 96-well plate containing 100 μl TSB with peptides (final concentration was 1/4 × MIC-1/16 × MIC). The plate was incubated at 37 °C for one hour without agitation to allow bacterial binding. Planktonic cells were then carefully removed by pipetting and the plate was washed thrice with 200 μl PBS solution. Then, 200 μl of 1.2% glutaraldehyde was added per well for 30 min for fixation and was then aspirated, and plate was allowed to air-dry. Adherent cells were stained for 2 min with 200 μl of 0.41% crystal violet (w/v in 12% ethanol) and washed thrice with PBS to remove extra dye. The wells were allowed to dry, and the crystal violet-stained well was re-solubilized with 200 μl of 95% ethanol and shaken for 10 min followed by measurement of the absorbance at 595 nm on a microplate reader (SpectraMax M5; Molecular Devices, Sunnyvale, CA, USA). The results were reported relative to untreated bacteria binding.

### Inhibition of biofilm formation

Biofilm formation assays were performed as described for the inhibition of bacterial initial attachment with some alternations. Plates containing peptides at serial dilutions and bacteria were incubated for 24 hours at 37 °C without agitation to allow biofilm formation and analysed after staining with 0.41% crystal violet as described above.

### Biofilm degradation assay

To determine whether the peptides could disperse preformed biofilms, *A. baumannii* biofilms were developed on MBEC pegs and then exposed to varying concentrations of peptides in fresh media for short time intervals. Briefly, overnight grown cultures of *A. baumannii* were diluted to an OD_600_ = 0.05 in fresh TSB, then 165 μl was transferred to the wells of a MBEC microtiter plate and the MBEC lid was placed on top of the wells. Biofilms were grown on the MBEC pegs with shaking for 24 hours. The lid was removed and transferred to a new plate with wells filled with AMPs to be tested. The pegs were immersed for 1 hour, and the lid was gently washed twice with 200 μl of PBS to remove non-adherent cells. Adherent biofilms on the pegs were fixed with 200 μl of 1.2% glutaraldehyde prior to staining for 2 min with 200 μl of 0.41% crystal violet. The pegs were washed several times with PBS to remove excess stain. Quantitative assessment of biofilm formation was obtained by the immersion of pegs in a sterile polystyrene microtiter plate that contained 200 μl of 95% ethanol. The plate was incubated at room temperature for 10 min with shaking, and the absorbance at 595 nm was determined using a SpectraMax M5 (Molecular Devices, Sunnyvale, CA, USA). The results were interpreted by the comparison of peptides on treated biofilms to untreated biofilms of *A. baumannii*. Experiments were performed in triplicate, and three independent experiments were performed for each of these assays.

## Additional Information

**How to cite this article**: Zhang, S.-K. *et al*. Design of an α-helical antimicrobial peptide with improved cell-selective and potent anti-biofilm activity. *Sci. Rep*. **6**, 27394; doi: 10.1038/srep27394 (2016).

## Supplementary Material

Supplementary Information

## Figures and Tables

**Table 1:** Sequence and biophysical properties of AR-23 and its analogues.

Peptide	Sequence	Calculated mass MW^a^	Observed mass MW^a^	Net charge	uH^b^	tR (min)^c^	Stabilization energy (kcal/mol)^d^
Melittin	GIGAVLKVLTTGLPALISWIKRKRQQ.NH_2_	2844.75	2845.4	6	0.499	19.14	−31.72
RV-23	RIGVLLARLPKLFSLFKLMGKKV.NH_2_	2625.68	2626.4	7	0.618	11.05	−29.63
AR-23	AIGSILGALAKGLPTLISWIKNR.NH_2_	2390.45	2391.3	4	0.548	16.6	−32.84
A(A1R)	**R**IGSILGALAKGLPTLISWIKNR.NH_2_	2475.52	2476.2	5	0.548	15.69	−24.31
A(A8R)	AIGSILG**R**LAKGLPTLISWIKNR.NH_2_	2475.52	2476.5	5	0.709	19.61	−26.52
A(I17K)	AIGSILGALAKGLPTL**K**SWIKNR.NH_2_	2405.46	2406.0	5	0.512	8.0	−29.83
A(I17R)	AIGSILGALAKGLPTL**R**SWIKNR.NH_2_	2433.45	2434.2	5	0.566	10.67	−23.18
A(A1R, A8R)	**R**IGSILG**R**LAKGLPTLISWIKNR.NH_2_	2560.58	2561.4	6	0.754	6.53	−25.73
A(A1R, I17K)	**R**IGSILGALAKGLPTL**K**SWIKNR.NH_2_	2490.53	2491.2	6	0.512	5.16	−27.54
A(A8R, I17K)	AIGSILG**R**LAKGLPTL**K**SWIKNR.NH_2_	2490.53	2491.5	6	0.704	6.59	−27.93
A(A1R, A8R, I17K)	**R**IGSILG**R**LAKGLPTL**K**SWIKNR.NH_2_	2575.59	2576.1	7	0.754	12.23	−31.72
A(A1R, A8R, I17R)	**R**IGSILG**R**LAKGLPTL**R**SWIKNR.NH_2_	2603.58	2604.4	7	0.754	10.78	−29.63

^a^Molecular weight (MW) as measured by mass spectroscopy (MS).

^b^μH, mean relative hydrophobic moment determined at website: http://www.bioinformatics.nl/emboss-explorer/.

^c^tR, retention time measured by reverse-phase HPLC.

^d^The stabilization energy of peptides was calculated using InsightII2000 software based on the computer-guided homology method.

**Table 2:** Circular dichroism data and ΔG_if_ of AR-23 and its analogues.

Peptides	% helix^a^			ΔG_if_ (kcal/mol)^b^	
	Water	30 μM SDS	50% TFE	30 μM SDS	50% TFE
Melittin	12	70	67	−8.03	−7.72
RV-23	5	39	33	−6.11	−5.56
AR-23	10	57	46	−8.82	−7.81
A(A1R)	8	49	38	−7.45	−6.44
A(A8R)	7	48	47	−7.36	−7.26
A(I17K)	5	43	32	−6.24	−5.22
A(I17R)	8	46	33	−6.69	−5.5
A(A1R, A8R)	7	40	34	−5.98	−5.43
A(A1R, I17K)	7	43	28	−5.6	−4.22
A(A8R, I17K)	8	45	30	−5.78	−4.4
A(A1R, A8R, I17K)	8	45	29	−5.14	−3.67
A(A1R, A8R, I17R)	6	41	32	−4.95	−4.12

^a^The helical content (in percentage) of a peptide was calculated with the following equation: α (%) = 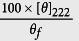
and *θ*_*f*_ = −39500 × 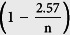, where α is the amount of helix and n is the number of amino acid residues, [*θ*_222_] is the experimentally observed absolute mean residue ellipticity at 222 nm.

^b^Calculations of ΔG_if_ according helix in 30 μM SDS and 50% TFE were performed with MPEx.

**Table 3:** Antimicrobial (MIC) and hemolytic (MHC) activities of AR-23 and its analogues against Gram-negative bacteria and human erythrocytes.

Peptides	MIC (μM)^a^			GM^b^ (μM)	MHC^c^ (μM)	TI^d^	Fold^e^
	*E. coli*	*P. aeruginosa*	*K. pneumoniae*				
melittin	12.5	6.25	6.25	7.87	0.78	0.1	
RV-23	6.25	6.25	6.25	6.25	6.25	1	
AR-23	25	12.5	12.5	15.75	3.12	0.2	1
A(A1R)	12.5	6.25	12.5	9.92	3.12	0.31	1.55
A(A8R)	12.5	12.5	12.5	12.5	3.12	0.25	1.25
A(I17K)	25	25	50	31.5	50	1.59	7.95
A(I17R)	12.5	12.5	25	15.75	25	1.59	7.95
A(A1R, A8R)	6.25	6.25	6.25	6.25	6.25	1	5
A(A1R, I17K)	25	12.5	25	19.84	100	5.04	25.2
A(A8R, I17K)	25	25	25	25	100	4	20
A(A1R, A8R, I17K)	12.5	12.5	12.5	12.5	200	16	80
A(A1R, A8R, I17R)	12.5	12.5	12.5	12.5	100	8	40

^a^Minimum inhibitory concentrations (MIC) were determined as the lowest concentration of peptide that prevented visible turbidity.

^b^The geometric mean (GM) of the peptide MICs against all four bacterial strains was calculated.

^c^MHC is the minimum haemolytic concentration that caused 10% haemolysis of human red blood cells (hRBC). When no detectable haemolytic activity was observed at 100 μM, a value of 200 μM was used to calculate the therapeutic index.

^d^Therapeutic index (TI) is the ratio of the MHC to the geometric mean of MIC (GM). Larger values indicate greater cell selectivity.

^e^Fold is the TI of analogue changes compared with AR-23.

**Table 4:** Antimicrobial (MIC) and hemolytic (MHC) activities of AR-23 and its analogues against Gram-positive bacteria and human erythrocytes.

Peptides	MIC (μM)^a^			GM^b^ (μM)	MHC^c^ (μM)	TI^d^	Fold^e^
	*S. aureus*	*S. epidermidis*	*B. subtilis*				
melittin	3.13	3.13	3.13	3.13	0.78	0.25	
RV-23	12.5	3.13	3.13	4.97	6.25	1.26	
AR-23	6.25	3.13	3.13	3.94	3.13	0.79	1
A(A1R)	6.25	3.13	6.25	4.96	3.13	0.63	0.8
A(A8R)	3.13	3.13	3.13	3.13	3.13	1	1.27
A(I17K)	50	12.5	6.25	15.75	50	3.17	4.01
A(I17R)	25	6.25	3.13	7.88	25	3.17	4.01
A(A1R, A8R)	12.5	3.13	6.25	6.25	6.25	1	1.27
A(A1R, I17K)	100	6.25	6.25	15.75	100	6.35	8.04
A(A8R, I17K)	100	6.25	12.5	19.84	100	5.04	6.38
A(A1R, A8R, I17K)	100	6.25	12.5	19.84	200	10.08	12.76
A(A1R, A8R, I17R)	100	3.13	12.5	15.76	100	6.35	8.04

^a^Minimum inhibitory concentrations (MIC) were determined as the lowest concentration of peptide that prevented visible turbidity.

^b^The geometric mean (GM) of the peptide MICs against all four bacterial strains was calculated.

^c^MHC is the minimum haemolytic concentration that caused 10% haemolysis of human red blood cells (hRBC). When no detectable haemolytic activity was observed at 100 μM, a value of 200 μM was used to calculate the therapeutic index.

^d^Therapeutic index (TI) is the ratio of the MHC to the geometric mean of MIC (GM). Larger values indicate greater cell selectivity.

^e^Fold is the TI of analogue changes compared with AR-23.
