# Supplementary material for: Design of an α-helical antimicrobial peptide with improved cell-selective and potent anti-biofilm activity
Source: Sci Rep. 2016 Jun 8;6:27394. doi: 10.1038/srep27394 (PMC4897634; doi:10.1038/srep27394)
Supplement: Supplementary Information [file srep27394-s1.pdf]

Design of an α-helical antimicrobial peptide with improved cell-selective and potent anti-biofilm activity

Shi-Kun Zhang[1], Jin-wen Song[1], Feng Gong[1], Su-Bo Li[1], Hong-Yu Chang[2], Hui-Min Xie[3], Hong-Wei Gao *[1], Ying-Xia Tan*[1], Shou-Ping Ji*[1]

1. Department of Tissue Engineering, Beijing Institute of Transfusion Medicine, Beijing, China.

2. Department of Paediatrics, General Hospital of the PLA Rocket Force, Beijing, China.

3. Department of Rehabilitation Center, General Hospital of the PLA, Beijing, China.

*Correspondence to: Shou-Ping Ji, Ying-Xia Tan and Hong-Wei Gao, Department of Tissue Engineering, Beijing Institute of Transfusion Medicine, 27 Taiping Road, Beijing 100850, China. E-mail: jishouping@yahoo.com; tuanhu333@126.com; gaohongwei1976@126.com

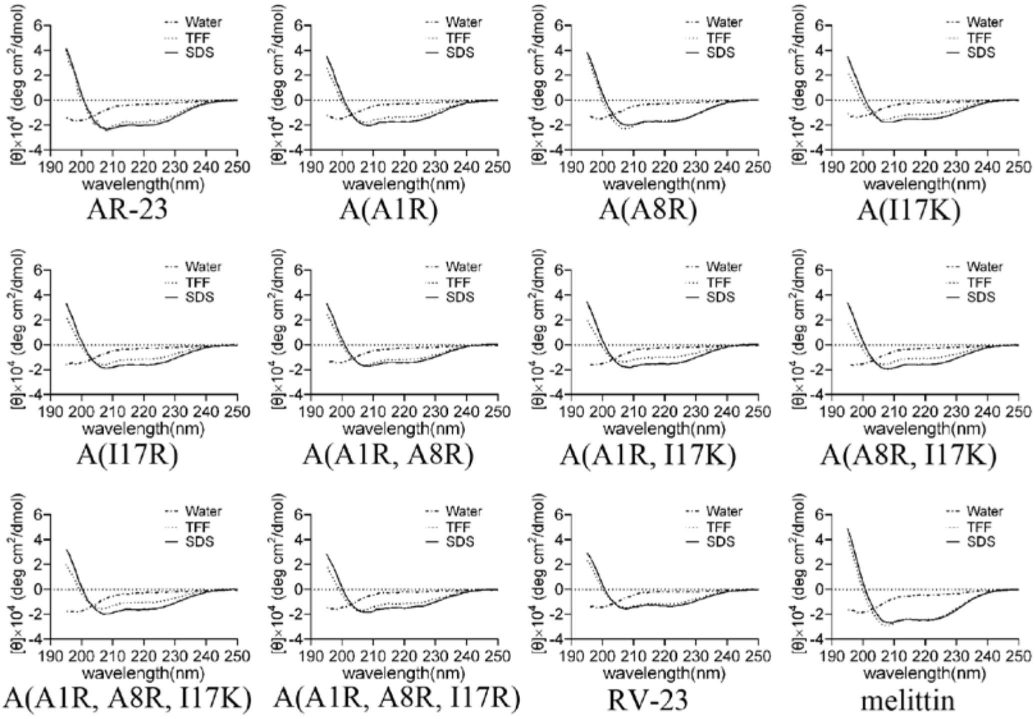

Figure S1 Determination of the secondary structures of peptide in water (dashed line), 30μM SDS (solid line) and 50%TFE (dotted line).

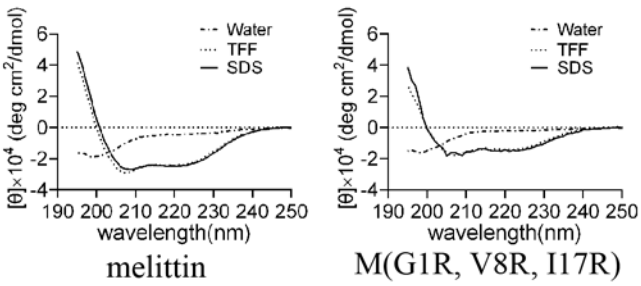

Figure S2 Determination of the secondary structures of melittin and M(G1R, V8R, I17R) in water (dashed line), 30μM SDS (solid line) and 50%TFE (dotted line).

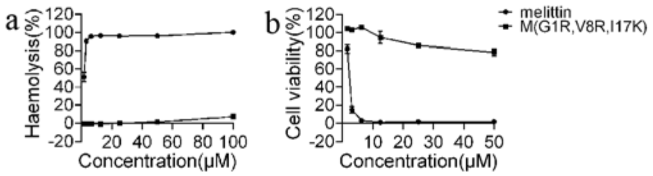

Figure S3 Toxicity activity of melittin and M(G1R, V8R, I17R). (a). Haemolysis activity of melittin and M(G1R, V8R, I17R) against hRBC. (b). Toxicity activity of melittin and M(G1R, V8R, I17R) against L929.

Table S1

Peptide	Sequence
Melittin	GIGAVLKVLTTGLPALISWIKRKRQQ.NH_2
M(G1R, V8R, I17R)	**R**IGAVLK**R**LTTGLPAL**K**SWIKRKRQQ.NH_2

Table S2

Peptides	MIC (μM)[a]			GM[b] (μM)	MHC[c] (μM)	TI[d]	Fold
	E.coli	*P.aeruginosa*	*K.pneumoniae*				
RV-23	6.25	6.25	6.25	6.25	6.25	1	
melittin	12.5	6.25	6.25	7.87	0.78	0.1	1
M(G1R, V8R, I17R)	25	25	12.5	19.8	200	10.1	101

[a] Minimum inhibitory concentrations (MIC) were determined as the lowest concentration of peptide that prevented visible turbidity.

[b] The geometric mean (GM) of the peptide MICs against all four bacterial strains was calculated.

[c] MHC is the minimum hemolytic concentration that caused 10% hemolysis of human red blood cells (hRBC). When no detectable hemolytic activity was observed at 100μM,a value of 200μM was used to calculate the therapeutic index.

[d] Therapeutic index (TI) is the ratio of the MHC to the geometric mean of MIC (GM). Larger values indicate greater cell selectivity.

Table S3

Peptides	MIC (μM)[a]			GM[b] (μM)	MHC[c] (μM)	TI[d]	Fold
	S. aureus	*S.epidermidis*	*B.subtilis*				
RV-23	12.5	3.125	3.125	4.96	6.25	1.26	
melittin	3.125	3.125	3.125	3.13	0.78	0.25	1
M(G1R, V8R, I17R)	100	6.25	3.125	12.5	200	16	64

[a] Minimum inhibitory concentrations (MIC) were determined as the lowest concentration of peptide that prevented visible turbidity.

[b] The geometric mean (GM) of the peptide MICs against all four bacterial strains was calculated.

[c] MHC is the minimum hemolytic concentration that caused 10% hemolysis of human red blood cells (hRBC). When no detectable hemolytic activity was observed at 100μM,a value of 200μM was used to calculate the therapeutic index.

[d] Therapeutic index (TI) is the ratio of the MHC to the geometric mean of MIC (GM). Larger values indicate greater cell selectivity.
